# Hepatic Arterial Therapy with Drug-Eluting Beads in the Management of Metastatic Pancreatic Carcinoma to the Liver: A Multi-Institutional Registry

**DOI:** 10.1155/2012/168303

**Published:** 2012-02-15

**Authors:** Raffi Kotoyan, Tiffany Metzger, Cliff Tatum, Ken Robbins, Robert C. G. Martin

**Affiliations:** ^1^Division of Surgical Oncology, Department of Surgery, University of Louisville School of Medicine, 315 East Broadway, Room 311, Louisville, KY 40292, USA; ^2^Norton Radiology, Norton Hospital, 301 South Floyd Street, Louisville, Ky 40202, USA; ^3^Baptist Health, Little Rock, AR 23011, USA

## Abstract

*Introduction*. There has been limited reporting on the use of hepatic-directed therapy in liver dominant hepatic metastases arising from pancreatic cancer. *Methods*. An IRB-approved prospective multi-institutional treatment registry of 885 patients undergoing 1458 treatments for primary or secondary cancers in the liver was evaluated from January 2007 to January 2011. *Results*. Ten patients underwent a total of 17 treatment sessions with drug-eluting beads (DEBs). Six patients received concurrent chemotherapy while undergoing DEB with no severe adverse events. After a median followup of 16 months, the 6- and 12-month response rates were 80% and 75%, respectively, with a median overall survival of 9.3 months. *Conclusion*. Hepatic arterial therapy with DEB can be safely and effectively used in selected patients with liver predominant metastatic disease from pancreatic cancer. This therapy should be considered in combination with systemic chemotherapy as a possible second therapy given the limited response rates of second-line chemotherapy.

## 1. Introduction

Pancreatic cancer is a major cause of cancer-related mortality worldwide as it ranks as the 5th leading cause of death, with an annual incidence in certain countries of approximately 20,000–40,000 cases and poor mortality rates [1]. Owning to the high frequency of local extension and/or metastatic disease at the time of diagnosis, only a small minority of patients are candidates for curative resection. Moreover, surgery alone is limited, with an unsatisfactory prognosis and a high incidence of postoperative recurrence. To improve the survival of patients with pancreatic cancer, effective multimodality treatments are demonstrating effective disease control and prolongation of quality of life time. A recent randomized controlled study demonstrated that treatment with gemcitabine exhibited a better clinical benefit response (CBR) (23.8 versus 4.8%) and median survival period (5.65 versus 4.41 months) than bolus 5-fluorouracil (5-FU) [2].

However, current chemotherapy regimens for pancreatic cancer must continue to be improved since gemcitabine alone offers limited survival benefit. Gemcitabine administration with a fixed dose-rate infusion [3] and gemcitabine combination regimens have been investigated, but a meaningful impact on survival, compared with that of gemcitabine monotherapy, has not been reached. Recent randomized phase III studies of gemcitabine plus erlotinib [4] and gemcitabine plus capecitabine [5] have demonstrated significant survival benefit, but a consensus on the optimal first line therapy has not been agreed upon.

Liver dominant and liver refractory metastatic diseases still remain common problems in the management of metastatic pancreatic cancer. Transcatheter arterial embolization (TAE) and transcatheter arterial chemoembolization (TACE) are increasingly used as regional therapeutic modalities for the treatment of unresectable hepatic malignancies [6]. In general, TAE and TACE have been used when surgical resection and/or systemic therapy have failed to produce an adequate response or when conventional therapy has been known to be ineffective [7]. There has been almost no information reported on the use of TACE in hepatic metastases arising from pancreatic cancer.

Drug-eluting bead TACE is a drug delivery system that combines the local embolization of vasculature with the release of chemotherapy into adjacent tissue. Its administration is similar to that of conventional TACE, and it represents a minimally invasive procedure performed by interventional radiologists [8, 9]. The beads occlude vasculature, causing embolization, and the chemotherapy is delivered locally. Early phase 1 and nonrandomized phase 2 studies have confirmed the ability of this device to deliver a local, controlled, sustained dose of doxorubicin to the tumors, with minimal systemic doxorubicin exposure [10]. A recently completed randomized phase 2 study demonstrated that these drug-eluting doxorubicin beads had improved response rates when compared to conventional TACE in advanced HCC and significantly less overall adverse events, including doxorubicin-related side effects [11]. Therefore, we conducted a prospective observational study to evaluate the safety and efficacy of hepatic arterial therapy in the management of liver dominant or liver refractory metastatic pancreatic cancer.

## 2. Materials and Methods

An IRB-approved prospective multi-institutional treatment registry of 805 patients undergoing 1358 treatments for primary or secondary cancers in the liver was evaluated from January 2007 to January 2011. Of the 805 patients, 10 patients presented with liver dominant metastatic pancreatic cancer to the liver and were treated with doxorubicin or irinotecan drug-eluting beads. The registry was designed to satisfy the strict criteria for critical appraising of the quality of a registry study with (1) a well-described patient population, (2) hypothesis generating and answering questions, (3) high quality data, with good quality control, (4) independent assessment of outcomes, (5) good clinically relevant followup with minimal loss of patients, and (6) comparable patient evaluation across all institutional participating [12].

Patients were included for therapy if they were 18 years of age or older, of any race or sex, had histologic and radiologic proof of metastatic pancreatic cancer to the liver by percutaneous biopsy, were able to give informed consent, and were eligible for treatment as previously described [13, 14]. Patients must have had an ECOG performance status score of less than or equal to 2 with a life expectancy of greater than or equal to 3 months, nonpregnant with an acceptable contraceptive in premenopausal women. Exclusion to therapy was contraindication to angiographic and selective visceral catheterization, significant extrahepatic disease, representing an eminent life-threatening outcome, greater than 75% of hepatic parenchymal involvement, severe liver dysfunction, pregnancy, and severe cardiac comorbidities. Only patients with liver dominant (defined as greater than 50% of the overall total disease burden) were considered for treatment.

Patients were followed for any treatment-related adverse experiences for 30 days after each treatment and monitored for survival for two years. Follow-up assessments included a triphase CT scan of the liver within at least one to two months from the treatment completion with the evaluation of the enhancement pattern of the target lesion and tumor response rates measured according to modified RECIST criteria [15].

 The decision to treat with DEB was based on either the need to dose reduce chemotherapy based on toxicity and maintain disease control or because of hepatic specific progression. Given that it is well established that pancreatic adenocarcinoma is a systemic disease, the use of concurrent chemotherapy and DEB was performed in all patients. The use of DEB in neuroendocrine (NET) tumors was based on the established disease biology and the extent of liver involvement.

## 3. Image-Guided Infusion Technique

Defining the amount of liver disease was integral to defining both the number of treatments and the type of catheter position and therapy that would be performed. For finite numbers of lesions, defined as less than four lesions, a treatment cycle was planned for a minimum of two dosing schedules of at least 100 mg of DEBDOX to 150 mg of DEBDOX [16, 17] loaded in two bead vials or 100 mg DEBIRI [18] loaded in one vial. Bead size of 100 to 300 microns was recommended. Treatment intervals are planned for every four to eight weeks. If toxicity and performance status changes during treatment, the intervals between DEB are extended. Repeat abdominal imaging every three months from the initial first treatment cycle is recommended to evaluate response as well as planned retreatment. A treatment cycle is defined as treatment of all liver disease. A treatment is hepatic arterial chemotherapy to one single lobe, which could also be a treatment cycle if a patient has only unilobar disease.

For diffuse disease, a plan of a minimum of four doses of 100 to 150 mg (depending on the extent of tumor burden and the extent of hepatic parenchyma reserve) is loaded into one or two bead vials of the similar size as above with the plan for at least two treatments per lobe with every three to four week dosing schedule, following toxicity, and extending the interval if toxicity was seen with planned repeat CT scan three months from the first dose to evaluate tumor response. For example, if patients present with bilobar disease, they would receive the first bead treatment to the right lobe, then three weeks after the second bead treatment to the left lobe, then three weeks later third bead treatment to the right lobe, and then again three weeks later to the left lobe. The reason for lobar infusion is based on the desire for drug delivery and less on inducing stasis in patients with multifocal lobar disease that is not amenable to superselective delivery. Additional embolic material is not recommended since prior studies have demonstrated the increase in adverse events but no benefit in response rates.

All bead therapies were performed with the DC/LC bead microspheres (Drug-Eluting Bead (DEB); http://www.biocompatibles.com/, Biocompatibles UK, Surrey, UK).

## 4. Results

Ten patients underwent a total of 17 treatment sessions with drug-eluting beads (DEBs) (Table 1). There were 6 females and 4 males with a median age of 69 years and a range of 45 to 77 years. All patients' cardiac and vascular medical histories were negative. 2 had a history of tobacco use with a median 40 pack per year history. Three had diabetes, 2 of which were insulin dependent. Only 2 of the patients had had prior cholecystectomies. The patients had a median Karnofsky Performance Scale of a 100%.

All 10 patients had a primary pancreatic tumor; 6 (60%) with an adenocarcinoma or 4 (40%) with a neuroendocrine tumor (NET). Six patients had a distinct number of hepatic lesions, and 4 patients had lesions which could not be quantified. Seven (70%) patients had <25% liver involvement, while 3 (30%) had 26–50% liver involvement.

Of the 10 patients, 2 (20%) had undergone a prior liver resection (Table 2). Seven (70%) patients (6 with adeno and 1 with NET) had had prior chemotherapy; 4 with Gemcitabine and 1 each with FOLFOX, FOLFIRI, and Doxorubicin/Streptozocin. In these 6 patients with adenocarcinoma, 5 had grade 3-4 either hematologic or neurologic toxicity that led to either a dose-delayed, dose-limiting toxicity or discontinuation of one of the chemotherapeutic agents. Four patients had local liver lesion disease progression with either new lesions or the growth of established lesions while on chemotherapy. Three patients had had prior trans-arterial chemoembolization (TACE); 2 with CAM and 1 with carboplatin, with only stable disease as best response and the reason for DEB therapy. Finally, 6 (60%) patients were undergoing concurrent chemotherapy, either to control small volume extrahepatic disease or based on the desire of the treating multidisciplinary team in order to obtain synergistic results with systemic chemotherapy and DEB therapy.

There were 17 total treatments, with a median of 1.5 bead courses per patient, ranging from 1 to 3 courses per patient (Table 3). All 17 (100%) of the treatments were considered a technical success. Pancreatic adenocarcinoma patients were all treated with Irinotecan beads, and NET patient were treated with doxorubicin beads. The total hepatic dose exposure for all the irinotecan beads was 250, with a range of 100 to 400, and 150 for the Doxorubicin beads with a range of 100 to 450. For 13 (76%) of the treatments, we utilized 100 to 300 micron beads, and, for the remaining 4 (24%) treatments, we chose to use 300 to 500 micron beads. There were minimal hematologic changes due to the treatments. Lab values showed an average decrease of 0.23 cells/*μ*L in white blood cells (range of −0.45–3.00 cells/ul). On average, a patient's hemoglobin increased by 0.8 g/dL (range −1.1–2.1 g/dL). In a complete evaluation of all hematologic parameters, there were no grade 2 or greater hematologic toxicities. We found only minimal changes in overall hematologic values as long as established bead size, technique, and dosages were utilized.

A total of 7 adverse events consisting of vomiting, pain, nausea, and gastritis were recorded (Table 4). None of these adverse events were grade 3 or higher, which was the cutoff for being considered a severe adverse event. The vomiting, pain, and nausea were consistent with postembolic syndrome and were found to be related more commonly to DEBIRI administration in the adenocarcinoma patients. The one NET patient reported grade 1 gastritis that was controlled with oral medication.

The patients were set to be followed at 3, 6, 9, 12, and 18 months. Their response rates were measured using a modified Response Evaluation Criteria in Solid Tumors (mRECIST) Criteria (Table 5). No patients died due to complications of the procedure within 90 days of the treatment. At 3 months (*N* = 10), 4 (40%) patients had a complete response to their treatment and 6 (60%) patients had a partial response. At 6 months, 2 patients had died due to their disease. Of the remaining 8 patients, 3 had a complete response. At the 9 month interval (*N* = 8), 2 (25%) patients had a complete response, 5 (63%) had a partial response, and 1 (13%) had stable disease. At the 12-month followup, 1 more patient had died of their disease. At this point (*N* = 7), 2 (25%) patients had stable disease, 2 (25%) had a complete response, and 3 (50%) had a partial response. Finally, at the 18 month followup (*N* = 6), 1 other patient had died of disease. Another patient's (*N* = 1) disease had progressed, 1 had a complete response, and 1 had stable disease. At the time of this writing, 4 patients had still not reached the time of the followup. After a median followup of 16 months, the 6- and 12-month response rates was 80% and 75%, respectively, with a median overall survival of 9.3 months.

## 5. Discussion

There remains limited information and limited clinical guidelines on the management of the subset of patients with liver predominant metastasis of pancreatic adenocarcinoma. However, by using what we know about treating similarly metastasized neuroendocrine tumors and other treatment modalities for adenocarcinoma, we hope to gain some better understanding of how to effectively treat patients with liver predominant metastatic pancreatic adenocarcinoma.

De Baere et al. demonstrated the safety and efficacy of DEBDOX with TACE to treat hepatic metastases from well-differentiated neuroendocrine tumors [19]. In this study, 20 patients underwent 34 sessions of TACE with DEBs loaded with Doxorubicin (500–700 micron beads). At 3 months, 80 percent of patients had a partial response, 3 patients had stable disease, and 1 had progressive disease. Patients had a median time of 15 months to progression of disease. In our study, no patients had signs of progression up to 12 months of follow-up. 67% of patients had postembolization syndrome which lasted less than 7 days, and 5 patients showed signs of TACE-induced peripheral liver necrosis at 1 month. It was concluded that TACE with DEBs was well tolerated and safe but needs more studies to define the best protocol to use such treatments.

Our study used a maximum of 300–500 micron size beads as compared to the 500–700 micron beads used in the study by De Baere et al. While all the patients in their study had metastatic neuroendocrine tumors, 4 out of 10 (40%) of our patients had neuroendocrine tumors, while the remaining 6 (60%) had primary pancreatic adenocarcinoma.

While the use of DEBDOX and DEBIRI in liver predominant metastases from pancreatic carcinoma is one modality to treat this condition, others have proven successful with a different approach using TACE. In a recent case report, Brown et al. examines the complete radiographic response in a patient with liver isolated metastases from pancreatic adenocarcinoma [20]. For this particular patient, conventional TACE with gemcitabine and cisplatin was used. This patient had a grade III poorly differentiated ductal adenocarcinoma that demonstrated hepatic metastasis on a follow-up CT at 14 months after recovery. The patient underwent chemoembolization with Gemcitabine and Cisplatin a total of three times. RECIST criteria were also used to evaluate this patient's response. At a 7-month followup, the patient showed no evidence of malignancy, and CT/PET showed no hypermetabolic foci.

Gemcitabine has been used systemically in the past for the treatment of pancreatic adenocarcinoma with only marginal activity [21]. The use of gemcitabine using TACE proved more efficacious in the case report by Brown et al. This increased response to liver-directed therapy may suggest a role for TACE in the treatment of liver dominant metastases from pancreatic adenocarcinoma.

Besides TACE, other treatments of metastatic pancreatic adenocarcinoma have been implemented and tested. Okusaka et al. have reported success using S-1, an oral fluoropyrimidine derivative in patients with metastatic pancreatic adenocarcinoma [22]. The patients treated in this study had inoperable pancreatic cancer and were administered a predetermined dose of S-1 based on their body surface area. In total, 144 treatments were administered to 40 patients. According to RECIST criteria, 0% of patients achieved complete response, 37.5% of patients achieved a partial response, while 32.5% of patients had progressive disease. The median time to progression was 3.7 months, and the median survival time was 9.2 months. This study had a larger patient population than our study, but we achieved a comparable median survival time of 9.3 months.

A limitation of this study may be that different doses and bead sizes were administered to each patient based on the physician's discretion. Also, our observations were based on a small patient population.

While there is much data on the treatment of metastatic neuroendocrine tumors, we hope to shed some light on the efficacy of our liver-directed therapy for metastatic pancreatic adenocarcinoma. Our study was based on a multi-institutional registry showing that the data is generalizable to any institution. While our patients showed some adverse effects, they were limited to vomiting, pain, and nausea and none were classified as severe. The limited toxicity profile of this treatment is beneficial for the patient with an already terminal illness. While the median survival of patients with metastatic pancreatic adenocarcinoma is 3–6 months [23], we achieved a median overall survival of 9.3 months. Future studies with reproducible results are needed to establish this treatment modality as the preferred form for metastatic pancreatic adenocarcinoma. We believe the results of our study show promise for its use in the future.

## Figures and Tables

**Table 1:** Clinical demographics for liver dominant metastatic pancreatic cancer DEBIRI-treated patients.

Characteristics	*N* = 10
Pancreatic primary	
Pancreatic adenocarcinoma Neuroendocrine	6 4

Age (years) (median, range)	(69, 45–77)

Body mass index (median, range)	23.8 (16.6–32.3)

Gender	
Male Female	4 6

Past medical history	
Cardiac Vascular Pulmonary Diabetes Insulin Non-insulin Alcohol Tobacco Median pack year smoking Hypertension Prior cholecystectomy	0 0 1 3 2 1 1 2 40 5 2
Karnofsky Performance Scale, median % (range)	100 (80–100)

Extent of liver lesions	
Distinct number Numerous	6 4

Liver involvement	
<25% 26–50%	7 3

Number liver tumors (median, range) 1 2 ≥3	(2, 1–3) 1 4 5

Sum of target lesion(s) size (median, range)	3.9 cm (1–10)

Extrahepatic disease Para-aortic lymph node Lung, adrenal Pancreatic primary	3 1 1 1

Lesion location	
Seg 2–4 Seg 4–8 Seg 5–8 Other	0 1 3 6

**Table 2:** Prior treatments.

*N* = 10 Patients	
Prior surgical therapy	
Liver resection	2

Prior chemotherapy	
FOLFOX FOLFIRI Gemzar Doxorubicin and Streptozocin	1 1 4 1

Prior interventional treatment	
RFA TACE—with CAM TACE—with carboplatin TAE—with particles	1 2 1 2

Concurrently chemotherapy	
FOLFOX Gemzar FOLFIRI Tarceva	2 2 1 1

**Table 3:** Bead catheter infusion outcomes.

	*N* = total treatments = 17
Number of bead courses	Median −1.5 (range) (1–3)

Technical success	100%

Dosage delivered (median, range)	(100, 25–150)

Total hepatic dose exposure for all LC Irinotecan Doxorubicin	Beads 250 mg (100–400) 150 mg (100–450)

Bead size utilized	
100–300 *μ* 300–500 *μ* 500–700 *μ*	13 4 0

Complications	4 (24%)

Extrahepatic infusion	0

Hematologic changes	
WBC HGB Bilirubin	−0.23 (−.45–3.0) 0.8 (−1.1–2.1) 0.3 (−0.4–0.8)

**Table 4:** Bead-infusion-related morbidity.

Adverse events (*n* = 7)	All grades	Severe grade*
Vomiting	2	1
Pain	1	1
Nausea	1	1
Gastritis	1	0
Other	2	0

*Defined as Grade 3 or higher.

**Table 5:** Response rates for all 10 patients evaluated*.

Response	3 mon *N* = 10	6 mon *N* = 8	9 mon *N* = 8	12 mon *N* = 8	18 mon *N* = 7
Complete response	4	3	2	2	1
Partial response	6	5	5	4	0
Stable disease	0	0	1	2	1
Progression of disease	0	0	0	0	1
Not reached time point	0	0	0	0	4
DOD	0	2	0	1	0
DOC	0	0	0	0	0

DOD: dead of disease; DOC: dead of complication.

*Response rates measured using modified RECIST Criteria.

## References

[1] Jemal A, Bray F, Center MM, Ferlay J, Ward E, Forman D (2011). Global cancer statistics. *CA Cancer Journal for Clinicians*.

[2] Burris HA, Moore MJ, Andersen J (1997). Improvements in survival and clinical benefit with gemcitabine as first- line therapy for patients with advanced pancreas cancer: a randomized trial. *Journal of Clinical Oncology*.

[3] Tempero M, Plunkett W, van Haperen VR (2003). Randomized phase II comparison of dose-intense gemcitabine: thirty-minute infusion and fixed dose rate infusion in patients with pancreatic adenocarcinoma. *Journal of Clinical Oncology*.

[4] Moore MJ (2005). Brief communication: a new combination in the treatment of advanced pancreatic cancer. *Seminars in Oncology*.

[5] Cunningham D, Chau I, Stocken DD (2009). Phase III randomized comparison of gemcitabine versus gemcitabine plus capecitabine in patients with advanced pancreatic cancer. *Journal of Clinical Oncology*.

[6] Liapi E, Geschwind JFH (2007). Transcatheter and ablative therapeutic approaches for solid malignancies. *Journal of Clinical Oncology*.

[7] Artinyan A, Nelson R, Soriano P (2008). Treatment response to transcatheter arterial embolization and chemoembolization in primary and metastatic tumors of the liver. *HPB*.

[8] Lewis AL, Taylor RR, Hall B, Gonzalez MV, Willis SL, Stratford PW (2006). Pharmacokinetic and safety study of doxorubicin-eluting beads in a porcine model of hepatic arterial embolization. *Journal of Vascular and Interventional Radiology*.

[9] Lewis AL, Gonzalez MV, Lloyd AW (2006). DC Bead: in vitro characterization of a drug-delivery device for transarterial chemoembolization. *Journal of Vascular and Interventional Radiology*.

[10] Poon RT, Tso WK, Pang RW (2007). A phase I/II trial of chemoembolization for hepatocellular carcinoma using a novel intra-arterial drug-eluting bead. *Clinical Gastroenterology and Hepatology*.

[11] Lammer J, Malagari K, Vogl T (2010). Prospective randomized study of doxorubicin-eluting-bead embolization in the treatment of hepatocellular carcinoma: results of the PRECISION V study. *CardioVascular and Interventional Radiology*.

[12] Levine MN, Julian JA (2008). Registries that show efficacy: good, but not good enough. *Journal of Clinical Oncology*.

[13] Martin RC, Robbins K, Tomalty D (2009). Transarterial chemoembolisation (TACE) using irinotecan-loaded beads for the treatment of unresectable metastases to the liver in patients with colorectal cancer: an interim report. *World Journal of Surgical Oncology*.

[14] Martin RC, Joshi J, Robbins K (2011). Hepatic intra-arterial injection of drug-eluting bead, irinotecan (DEBIRI) in unresectable colorectal liver metastases refractory to systemic chemotherapy: results of multi-institutional study. *Annals of Surgical Oncology*.

[15] Lencioni R, Llovet JM (2010). Modified recist (mRECIST) assessment for hepatocellular carcinoma. *Seminars in Liver Disease*.

[16] Joshi J, Robbins K, Válek V (2010). Transarterial chemoembolization with drug-eluting beads loaded with doxorubicin for the treatment of metastatic breast cancer to the liver: results from a multiinstitutional registry. *Journal of Interventional Oncology*.

[17] Brown RE, Gibler KM, Metzger T (2011). Imaged guided transarterial chemoembolization with drug-eluting beads loaded with doxorubicin (DEBDOX) for hepatic metastases from melanoma: early outcomes from a multi-institutional registry. *American Surgeon*.

[18] Martin RC, Howard J, Tomalty D (2010). Toxicity of irinotecan-eluting beads in the treatment of hepatic malignancies: results of a multi-institutional registry. *CardioVascular and Interventional Radiology*.

[19] de Baere T, Deschamps F, Teriitheau C (2008). Transarterial chemoembolization of liver metastases from well differentiated gastroenteropancreatic endocrine tumors with doxorubicin-eluting beads: preliminary results. *Journal of Vascular and Interventional Radiology*.

[20] Brown DB, Gonsalves CF, Yeo CJ, Witkiewicz AK, Carr BI (2010). One year survival with poorly differentiated metastatic pancreatic carcinoma following chemoembolization with gemcitabine and cisplatin. *Oncology Reports*.

[21] Casper ES, Green MR, Kelsen DP (1994). Phase II trial of gemcitabine (2,2'-difluorodeoxycytidine) in patients with adenocarcinoma of the pancreas. *Investigational New Drugs*.

[22] Okusaka T, Funakoshi A, Furuse J (2008). A late phase II study of S-1 for metastatic pancreatic cancer. *Cancer Chemotherapy and Pharmacology*.

[23] Chue BM (2009). Five-year survival of metastatic pancreatic carcinoma: a study of courage and hope. *Gastrointestinal Cancer Research*.

